# Citizen science as a tool for arboviral vector surveillance in a resourced-constrained setting: results of a pilot study in Honiara, Solomon Islands, 2019

**DOI:** 10.1186/s12889-021-10493-6

**Published:** 2021-03-16

**Authors:** Adam T. Craig, Nathan Kama, George Fafale, Hugo Bugoro

**Affiliations:** 1 University of New South Wales (Sydney), Sydney, New South Wales Australia; 2 Vector-borne Disease Unit, Solomon Islands Ministry of Health and Medical Services, Honiara, Solomon Islands; 3 Environmental Health Unit, Honiara City Council, Honiara, Solomon Islands; 4 Solomon Islands National University, Honiara, Solomon Islands

**Keywords:** Arboviral disease, Vector-borne disease, Dengue, Zika, Chikungunya, Surveillance, Citizen science, Community participation, Solomon Islands, Pacific

## Abstract

**Background:**

Recent arboviral disease outbreaks highlight the value a better understanding of the spread of disease-carrying mosquitoes across spatial-temporal scales can provide. Traditional surveillance tools are limited by jurisdictional boundaries, workforce constraints, logistics, and cost; factors that in low- and middle-income countries often conspire to undermine public health protection efforts. To overcome these, we undertake a pilot study designed to explore if citizen science provides a feasible strategy for arboviral vector surveillance in small developing Pacific island contexts.

**Methods:**

We recruited, trained, and equipped community volunteers to trap and type mosquitos within their household settings, and to report count data to a central authority by short-message-service. Mosquito catches were independently assessed to measure participants’ mosquito identification accuracy. Other data were collected to measure the frequency and stability of reporting, and volunteers’ experiences.

**Results:**

Participants collected data for 78.3% of the study period, and agreement between the volunteer citizen scientists’ and the reviewing entomologist’s mosquito identification was 94%. Opportunity to contribute to a project of social benefit, the chance to learn new skills, and the frequency of engagement with project staff were prime motivators for participation. Unstable electricity supply (required to run the trap’s fan), insufficient personal finances (to buy electricity and phone credit), and inconvenience were identified as barriers to sustained participation.

**Conclusions:**

While there are challenges to address, our findings suggest that citizen science offers an opportunity to overcome the human resource constraints that conspire to limit health authorities’ capacity to monitor arboviral vectors across populations. We note that the success of citizen science-based surveillance is dependent on the appropriate selection of equipment and participants, and the quality of engagement and support provided.

**Supplementary Information:**

The online version contains supplementary material available at 10.1186/s12889-021-10493-6.

## Background

Recent Zika, chikungunya, and dengue outbreaks [1–3] demonstrate that invasive mosquito species that are also disease vectors pose a significant threat to public health. Such species include *Aedes aegypti* and *Aedes albopictus* mosquitos, the primary vectors responsible for dengue, chikungunya and Zika virus transmission, and *Anopheles* mosquitos, responsible for malaria transmission. Climate and environmental change, the proliferation of global travel and trade, and increasing human mobility have meant that non-native species are increasingly introduced into new areas while native species push the boundaries of their ranges, all with implications for biodiversity, economic loss, and health outcomes [4–6].

Recent arboviral disease outbreaks [7–11] have highlighted the public health value a better understanding of the spread of disease-carrying mosquitoes across multiple spatial-temporal scales can provide. Traditional surveillance tools are limited by jurisdictional boundaries, logistics, and cost [4, 12]; factors that, in low- and middle-income countries, often conspire to undermine public health protection efforts.

In attempts to overcome these limitations, investigators have explored the role citizen science may play in disease-carrying mosquito surveillance, with encouraging results [13–19]. Citizen science can be defined as ‘the practice of amateur (or non-professional) participation in scientific research, including data collection, to aid scientific investigation and knowledge’ [20]. While these trials have been conducted in both developed and developing settings, to the best of our knowledge, no research exploring the utility citizen science may play as a tool for arboviral vector surveillance in a Pacific island context has been conducted.

The Pacific region covers one-third of the earth and is home to approximately 12-million people (excluding Australia and New Zealand). Of these, 8.3 million reside in Papua New Guinea, with the remainder dispersed over the many thousands of islands and atolls that make up the other 21 Pacific island countries and territories (PICTs) [12]. Arboviral outbreaks are common in the Pacific with several significant outbreaks affecting the region in recent years, [11, 21–25] including the largest dengue outbreak to affect the Pacific islands in record.

Here, we report the implementation and results of a pilot study designed to explore if citizen science provides a feasible strategy for arboviral vector surveillance in small developing Pacific island contexts. The research questions posed were: are participants (citizen scientists) able to perform mosquito surveillance tasks for an extended period? How accurately are citizen scientists able to identify mosquito types? And, what factors supported and inhibited participants’ ability to perform required surveillance-related tasks with rigor?

## Method

### Setting

**Fig. 1:**
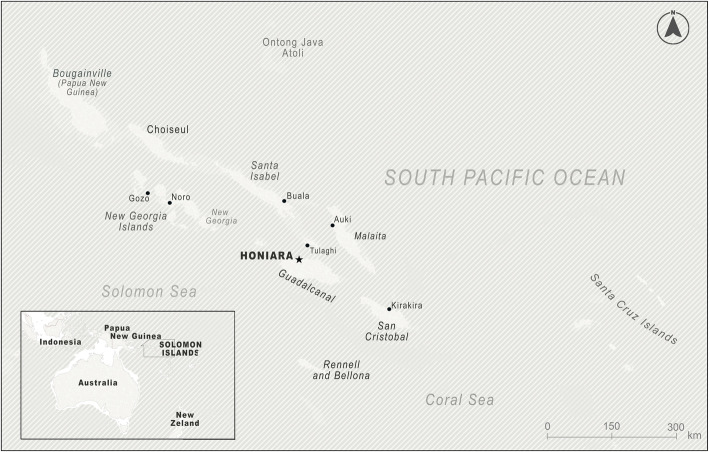
Map and location of Solomon Islands. The map was generated using licences obtained for ArcGIS Pro and Adobe Illustrator using open-source vector base maps available from Esri, the Food and Agriculture Organization of the United Nations, and the National Oceanic and Atmospheric Administration of the United States Department of Commerce

Solomon Islands is a low-income country ranked 153 of 189 nations in human development [26] located in the south-west Pacific Ocean, approximately 1800 km north-east of Australia (Fig. 1). The country’s population of 695,000 are dispersed over 992 islands and atolls; 78% reside in rural areas [27]. The pilot study was undertaken in Honiara, the capital of Solomon Islands. Honiara’s population was approximately 90,500 in 2019 [28].

### Participant recruitment

Thirteen participants were recruited using a snow-ball sampling method with potential candidates screened for eligibility using pre-determined criteria. To be eligible, participants had to be a resident of Honiara and intend to be in the city for the duration of the study period; have a secure outdoor area in which a mosquito trap could be set up; have a mobile phone (to send and receive short-message-service (SMS) messages); be available to attend a one-day training workshop; and be willing to collect and type mosquitos and submit surveillance data for the duration of the study. The number of participants was limited by funds available to support the pilot activities. On enrolment, data about participants’ age, sex, education level, home location, and previous experience with public health surveillance programs were collected.

### Equipment

Each participant was provided with a BG-Sentinel II trap (for mosquito collection), a magnifying glass and a pictorial mosquito identification card (Additional file 1) (to assist in mosquito species and genus identification), ridged containers (in which to place trapped mosquitos), and zip-lock bags, and data collection forms. While the BG-Sentinel II trap can be run from a battery (in addition to from mains power) and fitted with a carbon dioxide-emitting mosquito lure these additions was not used as it was unfeasible to do so, given logistical and project constraints. The mosquito identification card was developed based on existing resources [29, 30]. Participants were provided with written instructions and a small allowance to cover out-of-pocket expenses (e.g., travel to/for the training, SMS costs, electricity cost).

### Training

At a one-day workshop participants were taught how to set and empty the mosquito traps, neutralise caught mosquitos, identify mosquito to genus and species level, and record and report collected data. Mosquito identification was taught though lecture-style presentation and demonstration followed by practical activities that saw participants work with an entomologist to handle and type mosquito specimens.

Within 3-days of the workshop, a researcher visited participants at their homes to reiterate key information provided at the training, answer questions, and negotiate an appropriate location to place the trap.

### Mosquito data collection, reporting, and verification

Participants were required to identify and record the genus and species of trapped adult mosquitos and report these data by SMS to the research team on a weekly basis for an 8-week period. Data for the aggregate number of mosquitos trapped, stratified by mosquito genus/species, was reported. Feedback summarising the results of data that was reported was sent to participants by SMS each week.

Participants were required to place all trapped mosquitos in a single ridged container (that was provided) together with a completed data collection form for the corresponding period into a labelled zip-lock bag. These bags were collected and transported to the National Vector-borne Disease Control Laboratory where an entomologist independently (i.e., blind to the participants’ assessment) examined and typed the collected mosquitos. The weekly net numbers of each mosquito species and genus reported by the participant and the entomologist was recorded in a MS Excel® database. The agreement, expressed as a percentage, between participants’ and the entomologist’s assessment of mosquito type was then calculated.

### Qualitative data

At the completion of the data collection, semi-structured qualitative interviews (Additional file 2) were conducted with participants to solicit information about their experience as a citizen scientist, to identify challenges faced, and seek views on how the project, from a participant’s perspective, could be improved. Interview notes were kept, and the interviews audio recorded. AC and NK conducted the interviews. Interviews took approximately 30-min to complete.

### Analysis

Surveillance data were collated in MS Excel® and analysed using SPSS® v25 to measure participants’ ability to accurately detect and report catch data. The median and inter-quartile range of four metrics were calculated – the frequency, timeliness, completeness of participant reporting, and the agreement between participant and entomologist mosquito species and genus identification. Where appropriate, a Mann-Whitney U test was used to test for statistical differences between independent groups at a significance level of 0.05.

Qualitative data collected through interviews were analysed using a general inductive approach [31] to identify recurring and salient themes.

## Results

Thirteen citizens scientists (eight male and five female) were recruited into the pilot study. Ten had no previous experience with public health surveillance and three did. Participants’ ages ranged from 19 to 58 years, and four had completed tertiary education (Table 1).

**Table 1:** Summary information about citizen scientists and the surveillance data collected

	Any previous experience with public health surveillance			***p***-value
	Yes	No	Total	
Number	3	10	13	
Sex				
Male	3	5	8	
Female	0	5	5	
Age				
≤ 20 years	0	1	1	
21–40 years	1	3	4	
41–60 years	2	2	4	
Unknown	0	4	4	
Education				
Primary school	0	1	1	
Secondary school	1	4	5	
Tertiary	2	3	5	
Unknown	0	2	2	
**Proportion of days contributed data**				
Median (IQR) [%]	87.5 (57.1–100)	74.0 (28.6–100)	78.3 (28.6–100)	0.482
≤ 25%	0	2	2	
26–50%	0	2	2	
51–75%	1	1	2	
76–100%	2	5	7	
**Accuracy of mosquito identification (i.e., a**greement between citizen scientists and entomologist) Median (IQR) [%]				
*Ae.albopictus*	95.9 (69.0–100)	50 (25.2–75.0)	95.9 (66.0–100)	
*Ae.aegypti*	95.7 (89.3–100)	75 (50.0–100)	95.6 (83.3–100)	
*An.farauti*	100 (66.9–100)	66.7 (66.7–66.7)	100 (66.7–100)	
All	100 (82.1–100)	93.8 (77.5–99.4)	94.0 (78.0–100)	0.762

*IQR* Inter-quartile range

The median duration participants collected data was 44 days, or 78.3% (IQR: 28.6–100) of the study period. Participants with previous public health surveillance experience were more compliant (albeit not at a statistically significant level) than those that did not have experience (*p*= > 0.05) (Table 1). Four participants (all with no prior experience with public health surveillance) collected data for less than half of the days required; four participants (31%) collected and reported data for every day of the study period.

### Mosquitos identification

The median number of mosquitos trapped (of any species) per participant was 297 (IQR: 230.5–530.8). Of these, 10.7% were mosquitos with disease-carrying potential; *Ae.albopictus* (5.9%), *Ae.aegypti* (4%), and *An.farauti* (< 1%)).

The median agreement between citizen scientists’ and the entomologist’s assessment of species and genera was 94%, with a 6.2% (*p*= > 0.05) difference between the mean those that had and those that did not have previous experience with surveillance (Table 1).

**Fig. 2:**
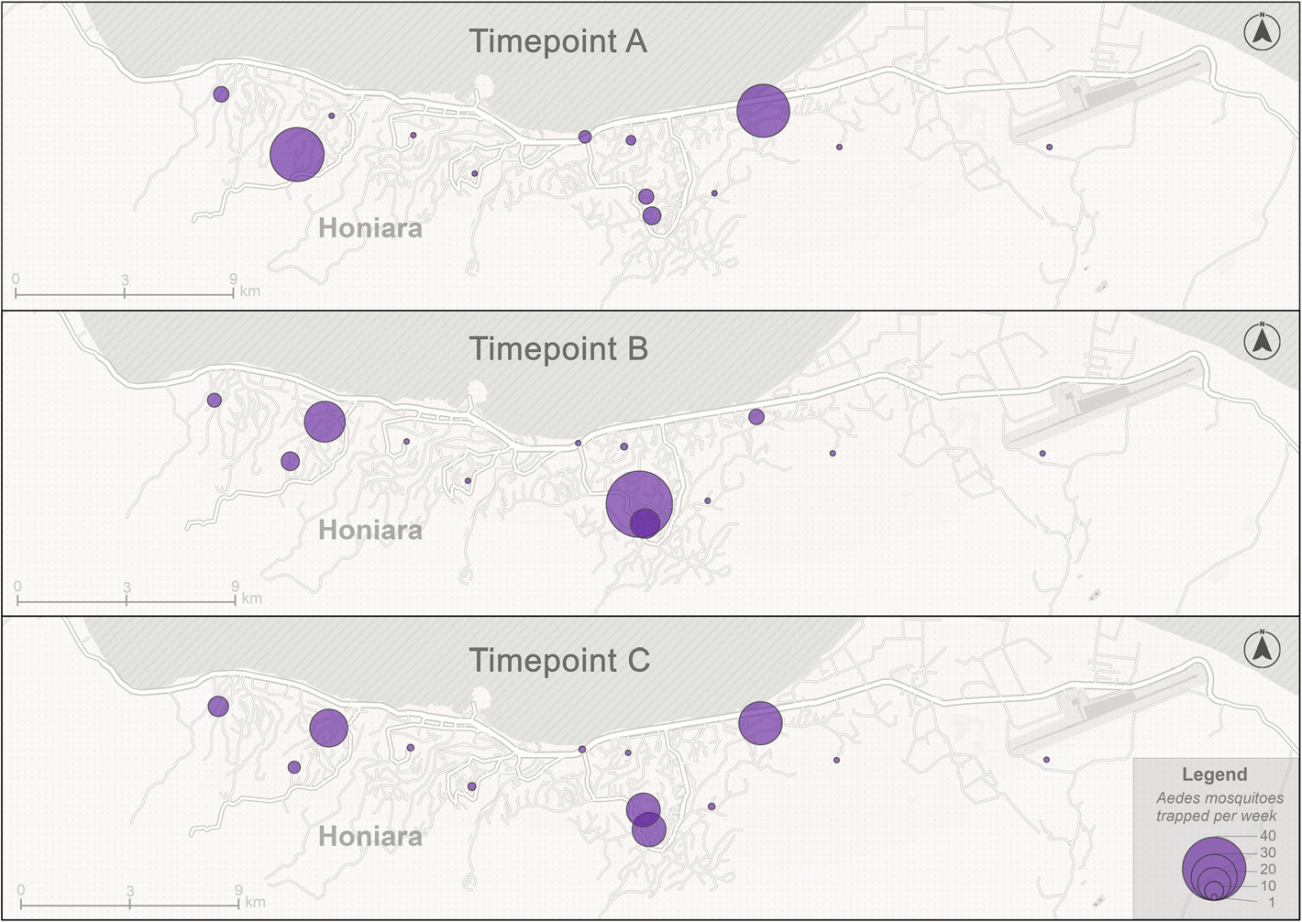
Example maps showing the number of *Aedes* mosquitos trapped by citizen scientists at three time points (week 1, week 5 and week 8 of the study). The map was generated using licences obtained for ArcGIS Pro and Adobe Illustrator using open-source vector base maps available from Esri, the Food and Agriculture Organization of the United Nations, and the National Oceanic and Atmospheric Administration of the United States Department of Commerce

The spatial distribution of mosquitos trapped varied over the 8-weeks of the pilot study, as shown in the example at Fig. 2.

### Enablers and barriers to citizen science-based mosquito surveillance

Ten end of program interviews with participants were conducted. Three participants were not contactable and hence not interviewed.

Overwhelmingly participants reported that the opportunity to contribute to a project of public health benefit was a key motivating factor for participation. One participant said, “I liked that I was doing something that told my family and community what the risks were, so we could then clean up and do something about it.” Another said, “it was great when we saw we caught more *Aedes* ones, we got the kids out to clean up the yard and empty out the pots so we wouldn’t get sick.”

Other motivating factors noted included the chance to learn new skills and to be provided with and use ‘scientific’ equipment. Regular engagement with a project officer (through SMS or face-to-face visits) was reported as the strongly motivating factor for ongoing participation in the project. Regular SMS messages received from the research team were said to be valued as they served as reminders to complete tasks required and reinforced the importance, currency, and relevance of the project for participants. “I really appreciated getting text messages about the project results. I’d see then and show my family how the numbers had changed,” a participant said.

We used BG-Sentinel II traps which required a power supply to operate. Our study found that some participants did not have access to a stable electricity supply, given the expense of electricity and frequency of brown- or black-outs. One participant reported, “While we received money [from the project] for electricity to run it [the trap], we often ran out [of electricity], this meant we were not able to collect mosquitos for a few days.” Another participant said, “My family saw the trap as a waste of our electricity and didn’t want it on all the time. They would turn it off when I was out”.

Trap placement was a concern for some households as the need to connect the trap to mains power meant a cord often had to be strung or laid across rooms, causing inconvenience, annoyance, and a trip hazard. Ensuring the trap was located securely away from animals and/or children was raised as a consideration.

Reasons reported for not participating included being too busy, being away for an extended period during the study, forgetting, or simply losing interest. Insufficient funds to purchase phone credit to send SMS reports to the research team was identified by some as a reason for not reporting. One participant commented, “I collected the data but could not send it until I got some money to buy ‘top-up’ [mobile phone credit].”

Finally, poor household lighting making mosquito identification difficult was identified as a barrier.

## Discussion

We report the results of a pilot study that aimed to determine if citizen science provides a feasible strategy for arboviral vector surveillance in a Pacific island developing state contexts. We found enthusiasm for the project which translated into high levels of engagement by most participants. We also found a high level of agreement between participant and entomologist assessment of the species and genus of trapped mosquitos suggesting that with basic equipment community members (citizen scientists) can identify mosquitos to the genus level with reasonable accuracy. These findings are encouraging and suggest that citizen science may offer a feasibility surveillance option for arboviral disease vector surveillance in the context. However, the pilot study identified operational barriers that may inhibit participation and undermine the stability and sustainability of citizen science approaches to mosquito surveillance, if left unchecked.

We used BG-Sentinel II traps, which require a power supply to operate. While considered high performing [32] our study found that the need for electricity to run these traps was problematic and that, perhaps, a simpler (and less expensive) trap, such as a Gravid Aedes Trap, would be more appropriate. Future implementation must weigh up the performance of a trap with the logistical requirements and cost to purchase and run it, and the associated surveillance system stability and sustainability implications incurred. This is particularly pertinent if citizen science-based surveillance approaches are to be implemented in rural and remote locations, where supplies of electricity are more likely to be unstable. Using a battery to run the BG-Sentinel II trap’s fan be an option, however cost and logistical challenges in doing so, and the threat this poses to surveillance system stability, must to be considered.

While we targeted and trained individuals, it was apparent that the tasks of trapping and typing mosquitos were often a shared household activity. This finding suggests that the success of citizen science implemented in community setting may be improved if groups of people (i.e., family units or social groups) are engaged. Further, the engagement and establishing sentinel sites within established community facilities (e.g., schools, clinics, churches) may allow implementors to leverage existing infrastructure (e.g., electricity supply) and human (and other) resources available within institutions to support sustainability and stability implementation of citizen science approaches.

Interestingly, several participants reported that the action of monitoring mosquito prevalence in their home environment motivated them to implement environmental and behavioural risk reduction measures. Further, participants expressed a keen interest in receiving feedback about the results of the project with SMS the preferred means of communication. These observations suggest that engagement of communities in practical surveillance activities that are perceived as having personal and/or community value may, in itself, be an effective health protecting strategy.

While SMS-based communication is an efficient tool for two-way communication, and may seem straight forward, it requires access to both a mobile phone and phone credit, often not available to citizens in Solomon Islands. Further, while the use of technology may offer convenience, it may also sever participant-to-project staff interaction which, we found, was a key factor for continued engagement.

We learned that the implementation of a citizen science project comes with notable administrative cost. We estimate that 1.5–2 days of staff time per week was consumed over the course of the pilot study. In the setting, this is a notable drain on staff resources that, if the program was to be sustained, would require dedicated funding. We note that much of the staff time was spent visiting households to collect specimen bags and entering data, activities that while important for this pilot study may not be required for scaled implementation of the surveillance method. Further, time and resources required to perform surveillance tasks may be reduced through the adoption of mobile phone-based mosquito species identification applications, such as iNaturalist (inaturalist.org) or Abuzz [33], and data transfer applications, such as Tupaia (tupaia.org). Similarly, alternatives to mosquito trapping and typing, such as mosquito egg collection and identification, [34, 35] may provide feasible alternatives for citizen science-based data collection. The impact out-of-pocket (to the participant) and health system costs may have on the feasibility and sustainability of citizen-science-based initiatives must be considered.

If these challenges can be addressed, by leveraging the human capital within communities citizen science-based approaches offer an opportunity to mitigate the resource and logistical constraints that impede surveillance practice. And in doing so the prospect of developing a more timely and better understanding of the spread of disease-carrying mosquitoes across spatial-temporal scales.

Our work is not without limitations. Notably, budget constraints limited the number of participants we were able to engage in the pilot study, and snowball sampling may have introduced a selection bias. While our results must be interpreted with caution our pilot work provides important and novel insights that will support those implementing (or considering implementing) citizen science-based model of mosquito surveillance.

## Conclusion

While there are challenges to address, our findings suggest that citizen science offers an opportunity to overcome the logistical and human resource constraints that conspire to limit health services’ capacity to monitor arboviral vectors across populations. We note that the success of citizen science-based surveillance is dependent on the appropriate selection of tools and participants, and the level and quality of engagement and support provided.

## Supplementary Information

**Additional file 1.** Simplified mosquito identification card.

**Additional file 2.** End of study participant interview data collection tool.

## Data Availability

The datasets generated and/or analysed during the current study are not publicly available due conditions of the study’s ethics approval but are available from the corresponding author on reasonable request.

## Abbreviations

*Ae.*: Aedes
*An.*: Anopheles
IQR: Inter-quartile range
MS: Microsoft
PICTs: Pacific island countries and territories
SMS: Short-Message Service
SPSS v25: Statistical Program for the Social Sciences, version 25
TDR: Special Programme for Research and Training in Tropical Diseases
WHO: World Health Organization
