# Supplementary material for: Citizen science as a tool for arboviral vector surveillance in a resourced-constrained setting: results of a pilot study in Honiara, Solomon Islands, 2019
Source: BMC Public Health. 2021 Mar 16;21:509. doi: 10.1186/s12889-021-10493-6 (PMC7962342; doi:10.1186/s12889-021-10493-6)
Supplement: Supplementary file 1 — Additional file 1. Simplified mosquito identification card. [file 12889_2021_10493_MOESM1_ESM.pdf]

SOLOMON ISLANDS CITIZEN SCIENCE SURVEILLANCE PROJECT, 2019

Simplified mosquito identification card

Aedes aegypti

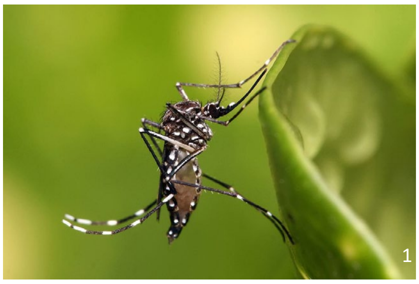

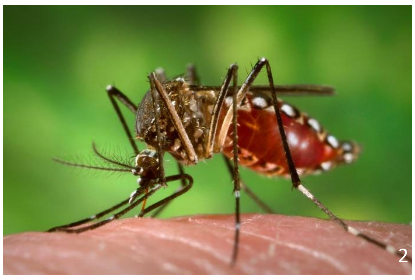

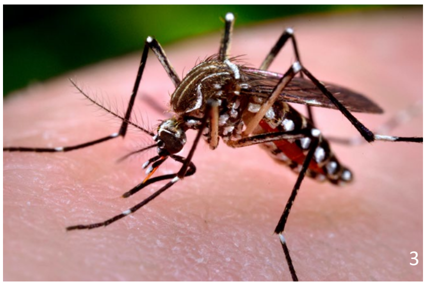

- Small to medium-sized mosquito, measuring approximately 4-7 mm in length
- White banding around their legs
- Double white stripes down the middle of their back (thorax).
- Wings without patched markings

Aedes albopictus

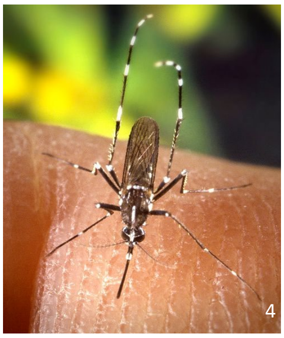

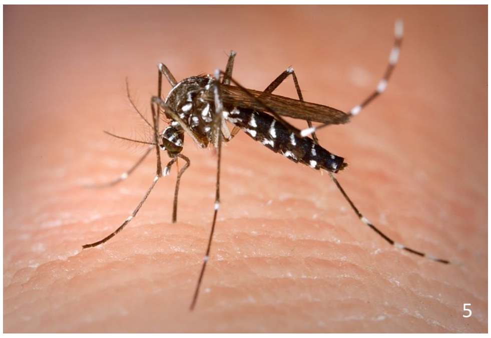

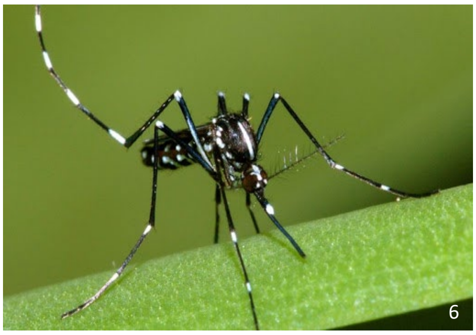

- Looks similar to the *Aedes aegypti* mosquito but is smaller (between 2-6 mm in length)
- White bands around their legs
- A single, silvery-white stripe down the middle of their back (thorax)
- Wings without patched markings

Anopheles farauti

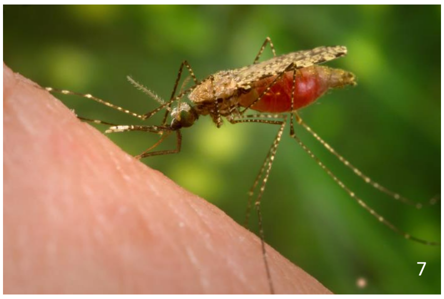

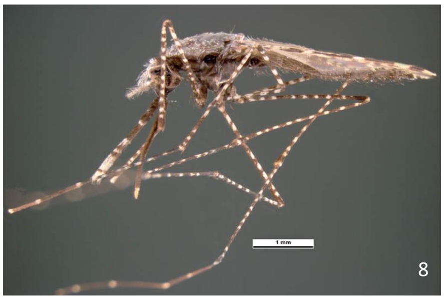

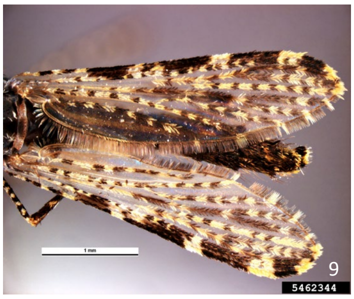

- Pale yellow-brown to dark brown coloured back
- Long and thin legs (no stripes)
- Wing often have patched markings

All images licenced as creative commons. Avail at: 1. commons.wikimedia.org/wiki/File:Aedes_aegypti.jpg; 2: commons.wikimedia.org/wiki/File:Aedes_aegypti_during_blood_meal.jpg; 3. commons.wikimedia.org/wiki/File:Aedes_aegypti_CDC-Gathany.jpg; 4. commons.wikimedia.org/wiki/File:Aedes_albopictus.jpg; 5. commons.wikimedia.org/wiki/File:Aedes_Albopictus.jpg; 6. commons.wikimedia.org/w/index.php…Aedes_albopictus_1.jpg; 7. https://scientistsagainstmalaria.net/parasites-vectors; 8. https://commons.wikimedia.org/wiki/File:Anopheles_farauti.jpg; 9. https://www.forestryimages.org/browse/detail.cfm?imgnum=5462344
