# Supplementary material for: Citizen science as a tool for arboviral vector surveillance in a resourced-constrained setting: results of a pilot study in Honiara, Solomon Islands, 2019
Source: BMC Public Health. 2021 Mar 16;21:509. doi: 10.1186/s12889-021-10493-6 (PMC7962342; doi:10.1186/s12889-021-10493-6)
Supplement: Supplementary file 2 — Additional file 2. End of study participant interview data collection tool. [file 12889_2021_10493_MOESM2_ESM.pdf]

Additional file 2: End of study participant interview data collection tool

**Administration**	
Location ID number (as per project register)	
Date of interview (dd/mm/yyyy)	
Interviewer(s) (Name(s))	
**Open-ended interview questions**	
1. To start, please tell me about your experience as a participant in this project?	
2. What motivated you to participate in the project?	
**3.** Were you able to perform data collection each week?	O Yes (1)<br>O No (2)
4. If not, why inhibited you?	
5. Did you have any issues with having the mosquito trap set up in your yard? If so, what were they?	
6. Can you talk to me about the training seminar you participated in at the beginning of the project, specifically whether or not it equipped you to undertake the tasks required?	
7. Looking back, was there anything missing from the training that, if covered, would have made your job as a citizen scientist easier?	
8. Did you have any problems setting and clearing the traps? If so, what were they?	
9. Tell me how you went with the task of identifying the different types of mosquitos caught in the trap? If you had any problems, could you please explain what they were?	
10. Please tell me about your experience with reporting data each week? I'd you had any problems, could you please explain what they were?	
11. Thinking about communication now. Do you have any throughs or comments about how the research team communicated with you? What changes, if any, would you suggest?	
12. Did you have any other issues or concerns you would like to raise?	
13. For you, what was the most rewarding aspect of being involved in the project?	
14. In your view, what could be done to make the tasks you were asked to perform easier?	
15. What value do you see in the program?	
16. Do you have any other comments that you would like to make?	
17. Would you be willing to participating in this project if it were to continue?	
